# Bilateral elevation of interleukin-6 protein and mRNA in both lumbar and cervical dorsal root ganglia following unilateral chronic compression injury of the sciatic nerve

**DOI:** 10.1186/1742-2094-10-55

**Published:** 2013-05-01

**Authors:** Petr Dubový, Václav Brázda, Ilona Klusáková, Ivana Hradilová-Svíženská

**Affiliations:** 1 Central European Institute of Technology (CEITEC), Masaryk University, Kamenice 3, Brno, 62500, Czech Republic; 2 Department of Anatomy, Division of Neuroanatomy, Faculty of Medicine, Masaryk University, Kamenice 3, Brno, 62500, Czech Republic; 3 Institute of Biophysics, Academy of Sciences of the Czech Republic, Královopolská 135, Brno, 61265, Czech Republic

**Keywords:** Unilateral nerve injury, Contralateral reaction, Remote ganglia, Neuroinflammation, Cytokines, Conditioning

## Abstract

**Background:**

Current research implicates interleukin (IL)-6 as a key component of the nervous-system response to injury with various effects.

**Methods:**

We used unilateral chronic constriction injury (CCI) of rat sciatic nerve as a model for neuropathic pain. Immunofluorescence, ELISA, western blotting and *in situ* hybridization were used to investigate bilateral changes in IL-6 protein and mRNA in both lumbar (L4-L5) and cervical (C7-C8) dorsal root ganglia (DRG) following CCI. The operated (CCI) and sham-operated (sham) rats were assessed after 1, 3, 7, and 14 days. Withdrawal thresholds for mechanical hyperalgesia and latencies for thermal hyperalgesia were measured in both ipsilateral and contralateral hind and fore paws.

**Results:**

The ipsilateral hind paws of all CCI rats displayed a decreased threshold of mechanical hyperalgesia and withdrawal latency of thermal hyperalgesia, while the contralateral hind and fore paws of both sides exhibited no significant changes in mechanical or thermal sensitivity. No significant behavioral changes were found in the hind and fore paws on either side of the sham rats, except for thermal hypersensitivity, which was present bilaterally at 3 days. Unilateral CCI of the sciatic nerve induced a bilateral increase in IL-6 immunostaining in the neuronal bodies and satellite glial cells (SGC) surrounding neurons of both lumbar and cervical DRG, compared with those of naive control rats. This bilateral increase in IL-6 protein levels was confirmed by ELISA and western blotting. More intense staining for IL-6 mRNA was detected in lumbar and cervical DRG from both sides of rats following CCI. The DRG removed from sham rats displayed a similar pattern of staining for IL-6 protein and mRNA as found in naive DRG, but there was a higher staining intensity in SGC.

**Conclusions:**

Bilateral elevation of IL-6 protein and mRNA is not limited to DRG homonymous to the injured nerve, but also extended to DRG that are heteronymous to the injured nerve. The results for IL-6 suggest that the neuroinflammatory reaction of DRG to nerve injury is propagated alongside the neuroaxis from the lumbar to the remote cervical segments. This is probably related to conditioning of cervical DRG neurons to injury.

## Background

Neuropathic pain has recently been defined as ‘pain arising as a direct consequence of a lesion or disease affecting the somatosensory system’ [1] and is therefore not directly associated with nociceptive input. Peripheral neuropathic pain, manifested as spontaneous pain and hyperalgesia, arises as a result of various forms of peripheral nerve damage, such as traumatic nerve injury, or neuropathy associated with diabetes or HIV infection [2,3].

There is compelling evidence indicating that hyperalgesia and ongoing pain due to peripheral nerve injury are associated with excitability of [4] and cellular and molecular changes in dorsal root ganglia (DRG), including proliferation and activation of satellite glial cells (SGC) [5], invasion of macrophages [6], and upregulation and downregulation of genes and proteins [7,8].

Pro-inflammatory and anti-inflammatory cytokines contribute to both induction and maintenance of neuropathic pain derived from cellular and molecular changes in the DRG [9,10]. Interleukin (IL)-6 is a member of the family of cytokines collectively termed ‘the interleukin-6-type cytokines’, which have diverse functions throughout the body. A growing body of evidence implicates IL-6 as a key component in the response of the nervous system to injury. For example, IL-6 is involved in promoting neuronal survival and protection against neuronal damage [11,12] and also in modulating pain [13,14].

In response to sciatic nerve transection, IL-6 protein and mRNA levels were found to be raised in medium to large sensory neurons 2 to 4 days after such damage in the ipsilateral, but not the contralateral, DRG homonymous to the injured nerve. By contrast, a nerve-constriction model induced lower concentrations of IL-6 protein and mRNA in DRG neurons, but both persisted longer than in a nerve-transection model. Presence of IL-6 in the nerve-constriction model of neuropathic pain correlated well with the duration of hypersensitivity [15,16].

A growing body of evidence indicates that unilateral nerve injury results in bilateral cellular and molecular changes in the nerve structures [17,18], and in bilateral changes indicated by behavioral tests [19]. In addition, neighboring uninjured DRG display changes after lesion of non-associated nerves [20,21].

The aim of the present study was to investigate quantitative alterations in IL-6 protein and mRNA levels following unilateral chronic constriction injury (CCI) of the sciatic nerve in both ipsilateral and contralateral DRG at L4-L5 and C7-C8 levels.

## Methods

### Animals and surgical procedures

Procedures were performed in accordance with protocols approved by the Animal Investigation Committee of the Faculty of Medicine, Brno, Czech Republic, and followed ethical guidance [22].

All experimental procedures were carried out under sterile conditions by the same person. The experiments were performed using 159 adult male Wistar rats 250–300 g in weight (Anlab, Brno, Czech Republic). The animals were housed on a 12 hour light/12 hour dark cycle at a temperature of 22 to 24°C, under specific pathogen-free conditions in the animal housing facility of Masaryk University. Sterilized standard rodent food and water were available *ad libitum*.

Surgical procedures were performed under deep anesthesia with a mixture of equal volumes of intraperitoneal (IP) ketamine 40 mg/ml and xylazine 4 mg/ml (Bioveta a.s., Czech Republic) (0.2 ml/100 g body weight). To prepare the unilateral CCI, the right sciatic nerve was exposed at mid-thigh level by blunt dissection just proximal to its trifurcation, and three ligatures (3-0 sutures; Johnson&Johnson, Ethicon, Inc., Belgium) were applied to reduce the nerve diameter by one-third. Animals who underwent CCI were left to survive for 1 (n = 21), 3 (n = 21), 7 (n = 21), or 14 (n = 21) days. The naïve control group consisted of 21 intact rats. Sham-operated rats (sham group; n = 54) had the right sciatic nerves exposed only, without lesion, and were allowed to survive for 1 (n = 21), 3 (n = 21), and 14 (n = 12) days. The assessors of the experimental groups were blinded to treatment (CCI versus sham) for all types of measurement.

### Behavioral tests

Withdrawal thresholds for mechanical hyperalgesia and latencies for thermal hyperalgesia were measured in both ipsilateral and contralateral hind and forepaws by dynamic plantar esthesiometer and plantar test (Ugo Basile, Italy), respectively. Rats were first acclimated in clear Plexiglas boxes for 30 minutes prior to testing. The paws were tested alternately with a 5 minute interval between tests. Six threshold and six latency measurements were taken for each paw during each test session 1 day before and 1, 3, 7, and 14 days after operation.

For thermal hyperalgesia, withdrawal time was measured and the intensity radiance was set to a value of 50. Data are expressed as mean ± SD of withdrawal thresholds (grams) and withdrawal latencies (seconds) for mechanical and thermal hyperalgesia, respectively.

### Immunohistochemical staining

Three naive rats and three rats for each period of survival from CCI (1, 3, 7, and 14 days) and sham operation (1, 3, and 14 days) were deeply anesthetized with a lethal dose of sodium pentobarbital (70 mg/kg body weight, IP) and perfused transcardially with 500 ml phosphate-buffered saline (PBS: 10 mmol/l sodium phosphate buffer, pH 7.4, containing 0.15 mol/l NaCl) followed by 500 ml of Zamboni’s fixative [23]. The L4-L5 and C7-C8 DRG from both sides were detected within their intervertebral foramina after total laminectomy and foraminotomy. The DRG were removed, immersed separately in Zamboni’s fixative at 4°C overnight, and then collected separately into samples of ipsilateral lumbar (L-DRGi), contralateral lumbar (L-DRGc), ipsilateral cervical (C-DRGi) and contralateral cervical (C-DRGc) DRG for each period of survival and each group of rats (naive, sham, and CCI). The samples were washed in 20% phosphate-buffered sucrose for 12 hours. Pairs of ipsilateral and contralateral DRG (C7-C8 or L4-L5 segments) were embedded in optimal cutting temperature compound (Tissue-Tek® OCT compound; Miles, Elkhart, IN USA) and cut together. Serial longitudinal cryostat sections (12 μm) through the DRG were mounted on chrome-alum coated slides, and processed for indirect immunohistochemical staining, which was performed simultaneously for lumbar and cervical segments.

**Interleukin-6 and interleukin-6 receptor immunofluorescence.** Sections were washed with PBS containing 0.05% Tween 20 (PBS-T) and 1% bovine serum albumin (BSA) for 10 minutes, treated with 5% normal donkey serum in PBS-T for 30 minutes, then incubated with 25 μl of rabbit polyclonal antibodies against IL-6 (1:500; Invitrogen Inc., Camarillo, CA, USA) or IL-6R (1:200; Santa Cruz Biotechnology, Santa Cruz, CA, USA) in a humid chamber at room temperature (21 to 23°C) for 12 hours. The immunohistochemical reaction was visualized by treatment with tetramethyl rhodamine isothiocyanate (TRITC)-conjugated and affinity-purified donkey anti-rabbit secondary antibody (1:100; Millipor, Billerica, MA, USA) for 90 minutes at room temperature. The control sections were incubated without the primary antibody or with the primary antibody saturated by recombinant rat IL-6 protein (Invitrogen). Sections were stained with Hoechst 33342 to detect positions of the cell nuclei, mounted in aqueous mounting medium (Vectashield; Vector Laboratories Inc., Burlingame, CA, USA) and analyzed using an epifluorescence microscope (DMLB; Leica Microsystems GmbH, Wetzlar Germany) equipped with a camera (DFC-480; Leica Microsystems) and a stabilized power supply for the lamp housing. The same immunostaining pattern for IL-6 and interleukin-6 receptor (IL-6R) was seen in the DRG of L4-L5 and of C7-C8 spinal-cord segments removed from the same side of naive, CCI and sham rats. Therefore, the results are described for the lumbar or cervical DRG of the ipsilateral or contralateral side.

**Double immunostaining.** Some of the sections taken through ipsilateral lumbar DRG from CCI rats surviving for 3 or 7 days were double-stained. After incubation with rabbit polyclonal anti–IL-6 antibody for 12 hours and intensive washing, the sections were covered with mouse monoclonal anti-glutamine synthase (anti-GS; 1:500; LS-C23895; LifeSpan BioSciences, Inc., Seattle, WA, USA), anti-CD68 (ED-1; 1:100; MCA341R) or anti-T-cell receptor (anti-TCR; 1:50; MCA453G) (both Serotec, Düsseldorf, Germany) antibodies and incubated for 4 hours. A mixture (1:1) of affinity-purified TRITC-conjugated donkey anti-rabbit and fluorescein isothiocyanate (FITC)-conjugated donkey anti-mouse secondary antibodies (Millipor, Billerica, MA, USA) was applied at a final dilution of 1:100 for 90 minutes at room temperature.

To visualize colocalization of IL-6 and activating transcription factor (ATF-3) indicating neuronal bodies with injured axons [24], the sections were incubated with monoclonal anti-IL-6 antibody (1:100; ARC0962; BioSource, Camarillo, CA, USA) and then rabbit polyclonal anti-ATF3 (1:200; sc188; Santa Cruz Biotechnology, Santa Cruz, CA, USA). After intensive washing, affinity-purified TRITC-conjugated donkey anti-mouse and FITC-conjugated donkey anti-rabbit secondary antibodies (Millipor, Billerica, MA, USA) were applied at a final dilution of 1:100 for 90 minutes at room temperature.

Sections incubated with rabbit polyclonal anti-IL-6R antibody for 12 hours were then treated with mouse monoclonal anti-GS for 4 hours. To visualize colocalization, the sections were incubated with TRITC-conjugated donkey anti-rabbit and FITC-conjugated donkey anti-mouse secondary antibodies at room temperature for 90 minutes.

### ELISA

Six naive rats and, CCI rats surviving for 1 (n = 6), 3 (n = 6), 7 (n = 6), and 14 (n = 6) days, and sham rats surviving for 1 (n = 6), 3 (n = 6), and 14 (n = 6) days were killed by CO_2_ inhalation. Blood samples were obtained by intracardiac puncture, and collected into tubes containing heparin and protease inhibitor cocktail (LaRoche, Basel, Switzerland). Plasma was immediately separated by low-speed centrifugation (2,500 *g* for 12 minutes).

Both ipsilateral and contralateral L4-L5 and C6-C7 DRG were removed and immediately collected in ice-cold PBS-T containing protease inhibitor cocktail (LaRoche, Basel, Switzerland). The DRG samples were divided into distinct groups of lumbar and cervical naive DRG (C-DRGn, L-DRGn) and ipsilateral and contralateral lumbar and cervical DRG (L-DRGi, L-DRGc, C-DRGi, and C-DRGc) taken from both CCI and sham rats for each period of survival. The DRG samples were homogenized in ice-cold PBS-T and separated by centrifugation (12,500 *g* for 12 minutes) to obtain extract proteins.

The tissue supernatant and plasma samples were stored at −60°C until analyzed. The total protein concentration was measured by spectrophotometer (Nanodrop ND-1000; Thermo Fisher Scientific Inc., Rockford, IL, USA) and the level of IL-6 protein was assessed by ELISA using a commercial kit with a sensitivity of 5 pg/ml (BioSource, Camarillo, CA, USA) in accordance with the manufacturer’s instructions. Each sample was measured five times using a microplate reader (SUNRISE Basic; Tecan, Salzburg, Austria) and data were standardized as pg of IL-6 protein to 100 μg of total protein. The IL-6 protein levels were normalized to baseline values of DRG and plasma from naive rats, which were set as 1, and final data are expressed as mean ± SD.

### Western blotting analysis

Naive rats (n = 6), CCI rats surviving for 1 (n = 6), 3 (n = 6), 7 (n = 6), and 14 (n = 6) days, and sham rats surviving for 1 (n = 6) or 3 (n = 6) days were deeply anesthetized with a lethal dose of sodium pentobarbital (70 mg/kg body weight, IP.). DRG of both sides were then detected within their intervertebral foramina after total laminectomy and foraminotomy. Whole DRG were extracted under aseptic conditions from L4-L5 and C7-C8 levels, and classified as ipsilateral lumbar (L-DRGi), contralateral lumbar (L-DRGc), ipsilateral cervical (C-DRGi), and contralateral cervical (C-DRGc) DRG for each period of survival and each group of rats (naive, CCI, and sham). These were fast-frozen in liquid nitrogen, then stored at −65°C until the time of analysis. For triplicate western blotting analysis, samples of DRG were collected from two rats in each group. The samples were homogenized in PBS containing 0.1% Triton X-100 and protease inhibitors (LaRoche) and separated by centrifugation at 10,000 *g* for 5 minutes at 4°C. The total protein concentration was measured in the tissue supernatant (Nanodrop ND-1000; Thermo Fisher Scientific) and normalized to the same levels. Proteins were separated by SDS-polyacrylamide gel electrophoresis [25] and transferred to nitrocellulose membranes by electroblotting (Bio-Rad Laboratories, Inc., Hercules, CA, USA). Blots were blocked by 1% BSA in PBS-T (3.2 mmol/l Na_2_HPO_4_, 0.5 mmol/l KH_2_PO_4_, 1.3 mmol/l KCl, 135 mmol/l NaCl, 0.05% Tween 20, pH 7.4) for 1 hours and incubated with anti-IL-6 polyclonal antibody (1:500; ARC0062; Biosource) overnight. Blots were washed in PBS-T and incubated with peroxidase-conjugated anti-rabbit IgG (1:1000; Sigma-Aldrich, St Louis, MO, USA) for 1 hour at room temperature. Equal loading of proteins was confirmed by α-tubulin staining. Protein bands were visualized using a chemiluminescence detection kit (ECL kit; Amersham Biosciences Inc., Piscataway, NJ, USA) on a chemiluminometer reader (LAS-3000; Bouchet Biotech) and analyzed using densitometry image software. After normalization to tubulin, IL-6 protein data were expressed as mean fold change relative to naive DRG, which was set as 1.

### *In situ* hybridization

For DRG tissue harvesting, three naive rats and three rats for each period of survival were deeply anesthetized with a lethal dose of sodium pentobarbital (70 mg/kg body weight, IP), and perfused transcardially with 500 ml PBS containing 0.1% diethylpyrocarbonate (DEPC), followed by 500 ml of 4% paraformaldehyde with 0.1% DEPC. The DRG samples were washed in 20% phosphate-buffered sucrose for 12 hours and embedded in OCT compound (Tissue-Tek®; Miles). Serial longitudinal sections 12 μm thick through the DRG were cut on a cryostat and then mounted on chrome-alum coated slides.

To localize the IL-6 gene transcript, *in situ* hybridization was performed in accordance with the protocol of Harnicarova and coworkers [26]. We used two 50-mer oligoprobes (VBC-Biotech, Vienna, Austria) synthesized for the target IL-6 gene transcript (Table 1). Digoxigenin (DIG)-dT was used for probe labeling. All solutions used in this procedure were prepared in double-distilled water treated with DEPC. DIG was detected with a commercial kit (DIG Colorimetric Nucleic Acid Detection Kit; LaRoche). The sections were mounted in aqueous mounting medium (Vectashield; Vector Laboratories) and analyzed using a microscope (DMLB; Leica Microsystems) equipped with a camera (DFC-480; Leica Microsystems). The control sections that had been incubated without the DIG-oligonucleotide probes displayed no color staining.

**Table 1:** **Probes used for *in situ *hybridization.**

**Probe**	**Sequence (5′→3′)**
1	CGCTGTTCATACAAT*CAGAATTGCCAT*TGCACAACTCT*TTTCTCATTTCC
2	TCAAGTGCTTTCAAGAT*GAGTTGGATGGTCTTGGT*CCTTAGCCACTCCTTC

Asterisks indicate position of digoxigenin labeling.

### Image analysis

The neuronal diameter, immunofluorescence intensity (brightness), and density of mRNA staining were assessed using an image analysis system (LUCIA-G; Laboratory Imaging Ltd., Prague, Czech Republic) in accordance with our previously published protocol [27]. Briefly, stained structures were detected for measurement after subtraction of background by the interactive thresholding technique (HSI: hue, saturation, and intensity) implemented in the image analysis (LUCIA) software, then transformed to binary mode. The binary foreground was monitored at every step of thresholding and manually edited if needed. The original color image was converted to gray and overlaid with the binary map. At least 200 neuronal profiles containing nuclei were measured for short (1 and 3 days) and late (7 and 14 days) periods of survival. The diameters of the DRG neurons were calculated from areas of neuronal profiles in sections for immunofluorescence and *in situ* hybridization, and the sizes of the DRG neurons were categorized as small (<25 μm), medium (25–40 μm), or large (>40 μm). The immunofluorescence and mRNA staining intensities were normalized to values of naive DRG and expressed as mean fold increase of intensity ± SD.

### Statistical analyses

Behavioral data were evaluated using Kruskal-Wallis one-way analysis with Bonferroni *post hoc* test and *P*<0.05 was considered significant. To verify differences in ELISA, a Bonferroni-corrected one-way ANOVA for repeated measures was run, with *P*<0.05 as the level of significant difference between tested samples. Data for naive and CCI rats for intensity of IL-6 and IL-6R immunostaining and density of IL-6 mRNA were tested using the Mann-Whitney U-test (*P*<0.05). All statistical analyses were performed using STATISTICA software (version 9.0; StatSoft Inc., Tulsa, OK, USA).

## Results

### Behavioral tests

**Figure 1:**
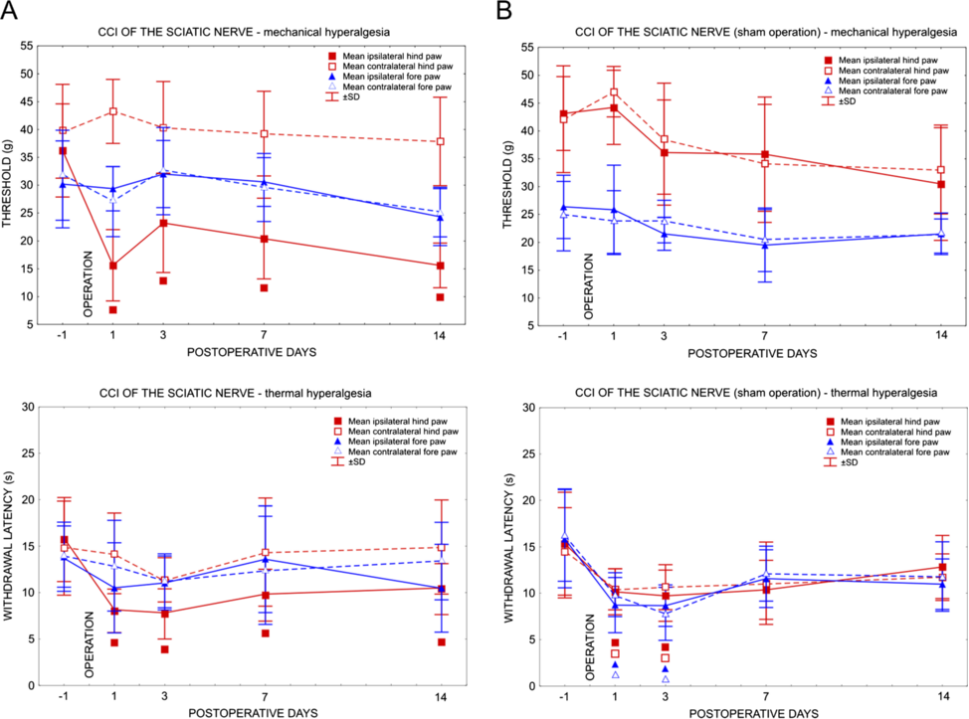
**Results of behavioral test.** (**A**) Results of behavioral tests in rats operated upon to create unilateral chronic constriction injury (CCI) of the sciatic nerve and (**B**) in sham-operated rats. Progressive development of evoked mechanical and thermal hyperalgesia was found in the ipsilateral hind paws of CCI rats. Transient thermal hyperalgesia was present bilaterally in both hind and fore paws 3 days after the sham operation. Data are expressed as mean ± SD of withdrawal thresholds (grams) and withdrawal latency (seconds) of mechanical and thermal hyperalgesia, respectively. Symbols identical to graph lines indicate significant differences (*P*<0.05) compared with measurements taken 1 day before operation.

All rats with CCI of the sciatic nerve displayed decreased thresholds of mechanical hyperalgesia and withdrawal latencies of thermal hyperalgesia restricted to the hind paws ipsilateral to nerve ligatures as signs of neuropathic pain. There were no significant changes in withdrawal threshold for mechanical and thermal hyperalgesia in the contralateral hind paws compared with the results 1 day before operation. Thresholds of mechanical and thermal hyperalgesia did not significantly change in either the ipsilateral or contralateral fore paws (Figure 1A).

Both ipsilateral and contralateral hind paws of sham rats exhibited a decrease in thresholds of mechanical hyperalgesia between days 3 and 14, but there was no statistical significance when compared with 1 day before surgical treatment. By contrast, significantly decreased withdrawal latencies of thermal hyperalgesia were found 1 and 3 days after the sham operation. This bilateral hypersensitivity for thermal stimuli was present in both fore and hind paws, and normalized during the following days of survival after the sham operation (Figure 1B).

### Immunohistochemical staining

#### Interleukin-6 immunofluorescence

**Figure 2:**
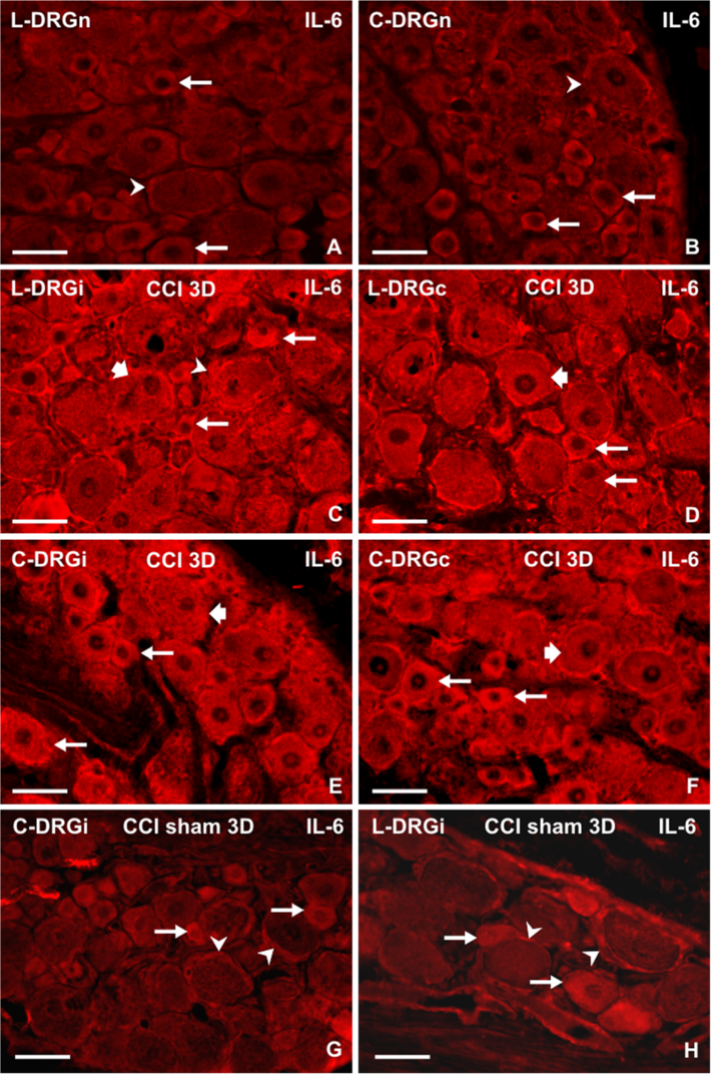
**Immunofluorescence staining for interleukin (IL)-6.** (**A-H**) Immunofluorescence staining for IL-6 in sections of dorsal root ganglia (DRG) removed from different groups of rats. Sections of (**A**) lumbar (L-DRGn) and (**B**) cervical (C-DRGn) DRG of naive rats. (**C,D**) Representative sections of L4 DRG from ipsilateral (L-DRGi) and contralateral (L-DRGc) sides of rats 3 days after unilateral chronic constriction injury (CCI) of the sciatic nerve (CCI 3D). (**E,F**) Cervical DRG from the ipsilateral (C-DRGi) and contralateral (C-DRGc) sides of the same rats (**G,H**) Ipsilateral cervical (C-DRGi) and lumbar (L-DRGi) DRG 3 days after sham operation. Large neurons (thick arrows), medium and small neurons (thin arrows), satellite glial cells (arrowheads). The sections were treated with rabbit polyclonal anti-IL-6 antibody and tetramethyl rhodamine isothiocyanate (TRITC)-conjugated donkey anti-rabbit secondary antibody under identical conditions. Scale bars = 60 μm.

Sections of lumbar and cervical DRG from naive rats showed weak immunofluorescence staining for IL-6 protein in small and medium-sized neuronal bodies and in SGC enveloping large neuronal bodies (Figure 2A,B). An increased intensity of IL-6 immunofluorescence was induced in all neuronal types of both ipsilateral and contralateral DRG removed from lumbar and cervical levels of all rats surviving up to 14 days after unilateral CCI of the sciatic nerve. A distinct increase in immunofluorescence intensity was particularly apparent in large neuronal bodies (>40 μm) compared with DRG removed from naive rats. The sections of lumbar and cervical DRG obtained from sham rats displayed a similar pattern and intensity of IL-6 immunofluorescence staining in the neuronal bodies to those removed from naive rats. In contrast to naive DRG, however, moderate intensity of IL-6 immunostaining was seen in SGC. Representative immunostaining for IL-6 in sections of lumbar and cervical DRG removed from both sides of rats surviving 3 days after CCI and sham operations is shown in Figure 2.

The control sections of DRG from CCI rats incubated with omission of the primary antibody or with the primary antibody saturated with protein were free of immunofluorescence staining (data not shown).

**Figure 3:**
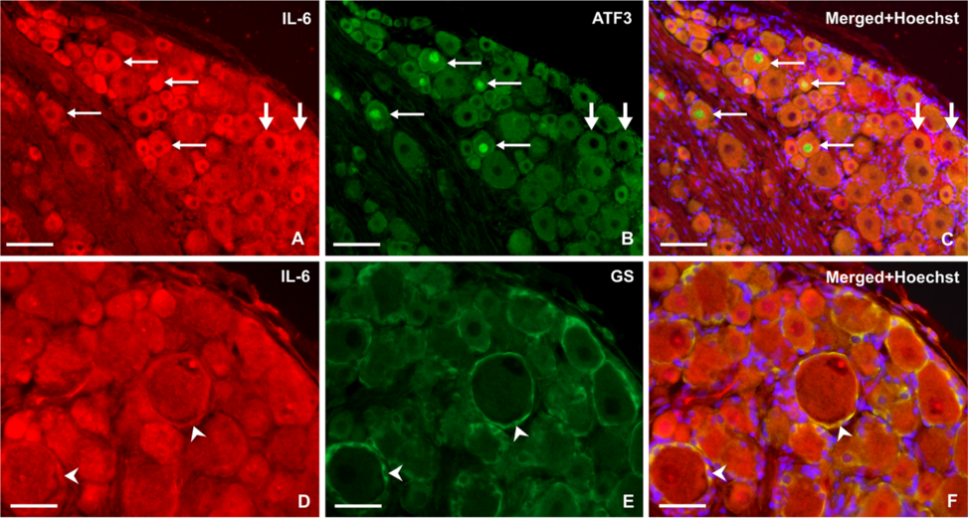
**Double immunofluorescence staining for interleukin (IL)-6 and activating transcription factor (ATF)-3 or glutamine synthase (GS).** Representative sections through ipsilateral lumbar DRG illustrating double immunostaining for (**A**,**D**) IL-6 and (**B**) ATF-3, indicating neuronal bodies with injured axons, and/or (**E**) GS as a marker of satellite glial cells (SGC). (**C**) Merged picture shows that increased IL-6 immunostaining was induced by chronic constriction injury (CCI) not only in ATF-3 positive neurons (thin arrows) but also in ATF-3 free neurons (thick arrows). (**F**) Merged picture shows an increase in IL-6 immunofluorescence in SGC (arrowheads) surrounding large neuronal bodies. Sections were cut through ipsilateral lumbar DRG of rats subjected to unilateral CCI of the sciatic nerve for 3 days. After incubation with mouse monoclonal anti–IL-6 and rabbit polyclonal anti-ATF-3, the section was covered with affinity-purified secondary antibodies: (**A**) tetramethyl rhodamine isothiocyanate (TRITC)-conjugated donkey anti-mouse; (**B**) fluorescein isothiocyanate (FITC)-conjugated donkey anti-rabbit. To identify IL-6 upregulation in SGC, the section was covered with rabbit polyclonal anti–IL-6 antibody and mouse monoclonal anti-GS. Affinity-purified secondary antibodies were applied: (**D**) TRITC-conjugated donkey anti-rabbit; (**E**) FITC-conjugated donkey anti-mouse. Positions of the cell nuclei were detected by staining with Hoechst 33342. Scale bars: (**A–C**) 60 μm; (**D–F**) 40 μm.

**Figure 4:**
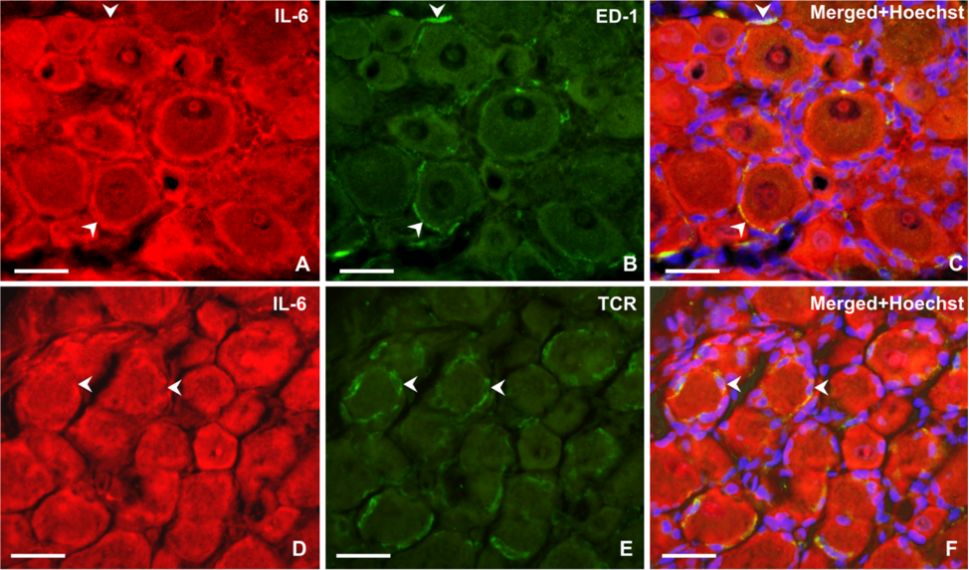
**Double immunofluorescence staining for interleukin (IL)-6 and ED-1 or T-cell receptor (TCR).** Representative sections through ipsilateral lumbar dorsal root ganglia (DRG) illustrate double immunostaining (**A,D**) for IL-6 and (**B**) ED-1, indicating invaded macrophages, and/or (**E**) TCR, a common marker of T cells. (**C,F**) Merged pictures show that ED-1+ macrophages and T cells (arrowheads) may contribute to IL-6 protein increase in lumbar DRG after chronic constriction injury (CCI) of the sciatic nerve. Sections were cut through ipsilateral lumbar DRG of rats subjected to unilateral CCI of the sciatic nerve for 3 days. After incubation with (**A,D**) rabbit polyclonal anti–IL-6 and (**B**) mouse monoclonal anti-ED-1 or (**E**) anti-TCR, the sections were treated with affinity-purified antibodies: (**A,D**) tetramethyl rhodamine isothiocyanate (TRITC)-conjugated donkey anti-rabbit and (**B,E**) fluorescein isothiocyanate (FITC)-conjugated donkey anti-mouse. Positions of the cell nuclei were detected by staining with Hoechst 33342. Scale bars: 60 μm.

Immunofluorescence for ATF-3, indicating neuronal bodies with injured axons [24], was found in neuronal nuclei of lumbar DRG ipsilateral to CCI but not in cervical DRG. Only a few neurons of contralateral lumbar DRG displayed ATF-3 immunostaining. The increased IL-6 immunoreactivity in ipsilateral lumbar DRG was present not only in ATF-3-positive but also in ATF-3-free neurons (Figure 3A–C). Double immunostaining also confirmed an increase in IL-6 protein in SGC (Figure 3D–F), and provided evidence of a contribution of ED-1+ macrophages and TCR+ cells to the IL-6 level in DRG removed from rats subjected to CCI (Figure 4).

**Figure 5:**
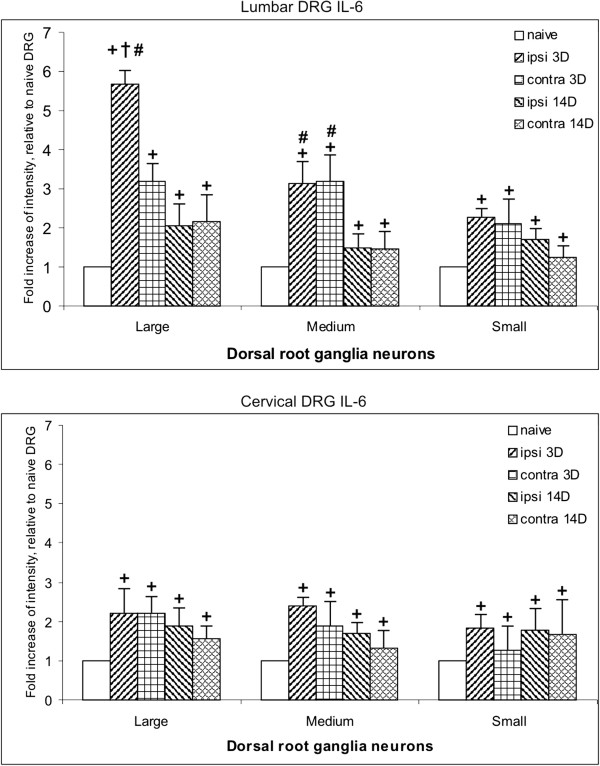
**Results from image analysis of interleukin (IL)-6 immunofluorescence intensity in dorsal root ganglia (DRG).** Results of immunofluorescence intensity for IL-6 in lumbar and cervical DRG removed from naive rats and those subjected to chronic constriction injury (CCI) for short (3 days) and long (14 days) periods of survival. Measurement by an image analysis system was carried out in large, medium, and small neuronal bodies of ipsilateral (ipsi) and contralateral (contra) DRG. Significant difference (*P*<0.05) compared with: +baseline level of naive rats, †value of contralateral counterparts, and #value between 3 and 14 days of survival.

Compared with naive DRG, a significant increase in IL-6 immunofluorescence intensity was detected in large, medium, and small neuronal bodies of both lumbar and cervical DRG from rats surviving for short (3 days) and long (14 days) periods after CCI treatment. The highest increase in IL-6 intensity was found in large neurons of lumbar DRG ipsilateral to CCI, and this level was significantly higher than the contralateral counterpart. However, the large neurons of cervical DRG and the medium and small neurons of both cervical and lumbar DRG displayed increased immunofluorescence intensity without significant differences when the ipsilateral and contralateral sides were compared in each period of survival. The IL-6 intensity of large neurons of ipsilateral lumbar DRG was significantly reduced when we compared short and long periods of survival, whereas a bilateral decrease was found in medium neurons for the same comparison. Small neurons in lumbar DRG and all types of neurons in cervical DRG did not display significant changes when short and long periods were compared (Figure 5).

#### Interleukin-6 receptor immunofluorescence

**Figure 6:**
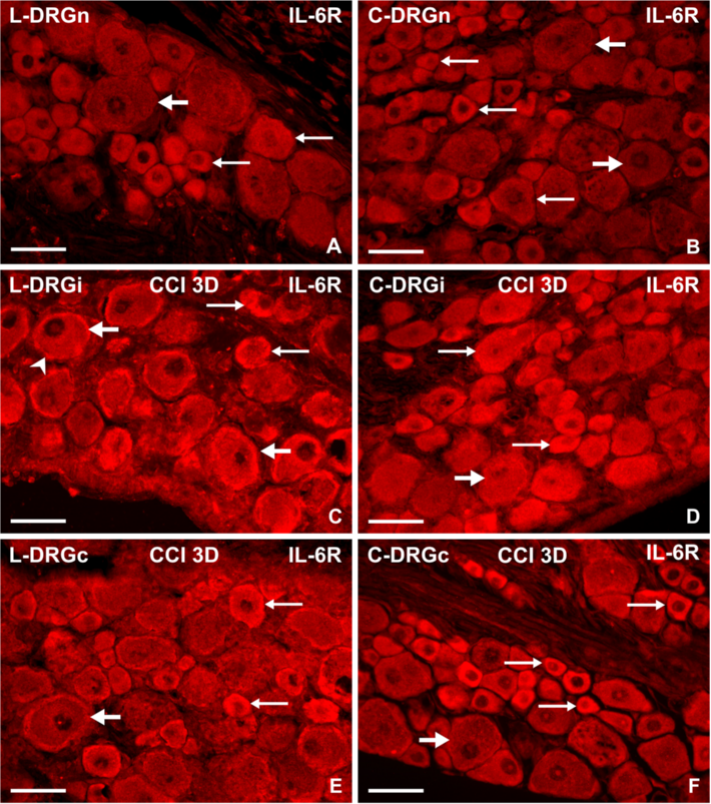
**Immunofluorescence staining for interleukin-6 receptor (IL-6R).** Representative sections of (**A,C,E**) lumbar and (**B,D,F**) cervical dorsal root ganglia (DRG) incubated for IL-6R immunohistochemical staining. Sections of L4 and C7 DRG removed from (**A,B**) naive rats and (**C**–**F**) from rats 3 days after chronic constriction injury (CCI) of the sciatic nerve. Immunofluorescence staining for IL-6R was increased in the large (thick arrows), medium, and small (thin arrows) neurons of lumbar and cervical DRG from both ipsilateral (DRGi) and contralateral (DRGc) sides of CCI rats compared with naive rats (DRGn). Increased IL-6R immunofluorescence was also present in satellite glial cells (SGC) of lumbar DRG ipsilateral to CCI (**C**, arrowhead). The sections were treated with rabbit polyclonal anti-IL-6R antibody and tetramethyl rhodamine isothiocyanate (TRITC)-conjugated donkey anti-rabbit secondary antibody under identical conditions. Scale bars = 80 μm.

**Figure 7:**
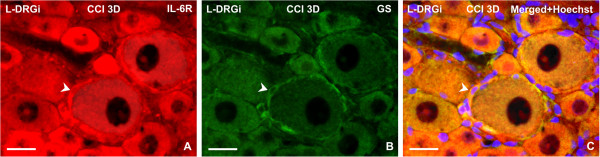
**Double immunofluorescence staining for interleukin-6 receptor (IL-6R) and glutamine synthase (GS).** Representative sections through lumbar dorsal root ganglia (DRG) incubated (**A**) with rabbit polyclonal anti-IL-6R and then (**B**) with mouse monoclonal anti-GS antibodies. To visualize colocalization, the section was treated with affinity-purified secondary antibodies: tetramethyl rhodamine isothiocyanate (TRITC)-conjugated donkey anti-rabbit and fluorescein isothiocyanate (FITC)-conjugated donkey anti-mouse. Staining with Hoechst 33342 was used to detect positions of the cell nuclei. (**C**) Merged picture shows increased immunofluorescence staining for IL-6R in satellite glial cells (SGC), indicated by simultaneous immunostaining for GS (arrowheads). Sections were cut through ipsilateral lumbar DRG of rats 3 days after unilateral chronic constriction injury (CCI) of the sciatic nerve. Scale bars = 40 μm.

**Figure 8:**
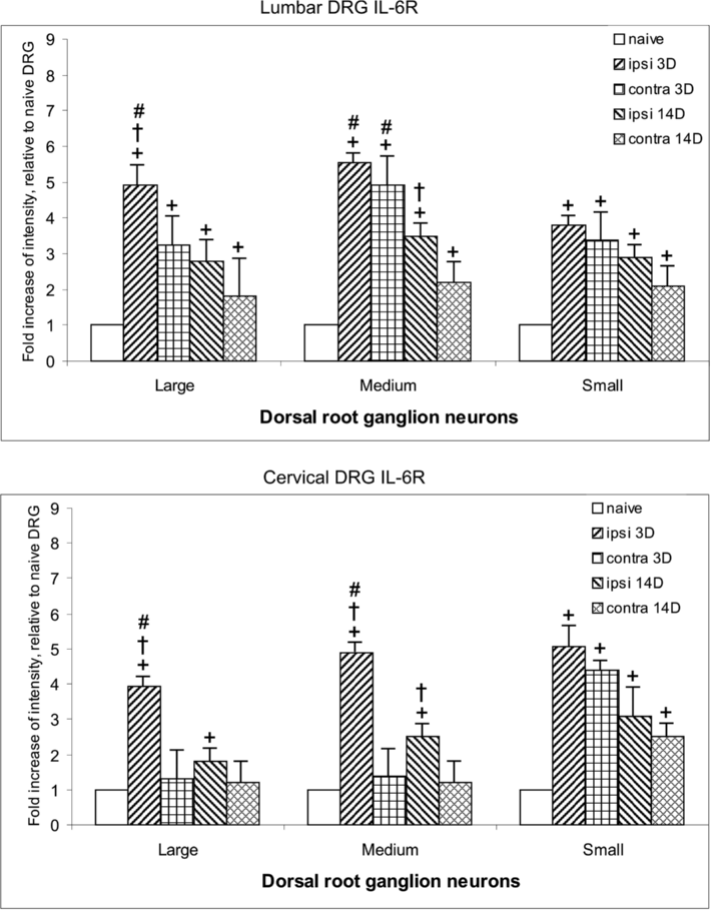
**Results of image analysis of interleukin-6 receptor (IL-6R) immunofluorescence intensity in dorsal root ganglia (DRG).** Results of immunofluorescence intensity for IL-6R in lumbar and cervical DRG removed from naive rats and those subjected to chronic constriction injury (CCI) for short (3 days) and late (14 days) periods of survival. Measurement by an image analysis system was carried out in large, medium, and small neuronal bodies of ipsilateral (ipsi) and contralateral (contra) DRG. Significant difference (*P*<0.05) compared with +baseline level of naive rats, †value of contralateral counterparts, and #value between 3 and 14 days of survival.

Sections of naive lumbar and cervical DRG displayed IL-6R immunofluorescence of moderate intensity in medium and small neurons, whereas large neuronal bodies had very weak or no immunostaining. Compared with naive DRG, there was a significant increase in IL-6R immunofluorescence bilaterally in all neurons of lumbar DRG from CCI rats for short (3 days) and long (14 days) periods of survival. Distinct immunofluorescence was also present in SGC, surrounding mainly large neurons of ipsilateral lumbar DRG (Figure 6). This was confirmed by double immunostaining for IL-6R and with GS used as a marker for SGC (Figure 7). There was a significantly increased intensity of IL-6R immunostaining in the neuronal bodies of all diameters in both lumbar and cervical DRG of CCI rats after 3 and 14 days (Figure 8). A significantly higher level of IL-6R immunofluorescence was found in the large neurons of lumbar DRG from the ipsilateral compared with the contralateral side, but no significant differences were found between the ipsilateral and contralateral medium and small neurons 3 days after CCI. The level of IL-6R immunofluorescence was significantly decreased in all types of neurons 14 days after CCI.

Neuronal bodies of all diameters also displayed significant increases in IL-6R immunofluorescence in the cervical DRG of the ipsilateral side compared with naive neuronal bodies. This increased immunostaining in ipsilateral cervical DRG was found at 3 and 14 days from CCI. The level was significantly higher in large and medium-sized neurons in the ipsilateral compared with the contralateral cervical DRG. By contrast, the small neurons displayed bilateral elevation of IL-6R immunostaining after both short and long periods of survival.

### ELISA assessment of interleukin-6 protein in dorsal root ganglia and plasma

The baseline level of IL-6 protein was significantly higher in cervical (124.9 ± 8.1 pg/0.1 mg total protein) than lumbar (75.1 ± 6.0 pg/0.1 mg total protein) DRG of the naive rats. Thus, the differences in DRG of different spinal levels should be borne in mind when comparing results of operated animals. Therefore, the IL-6 protein levels were normalized to the baseline values of the naive rats to compare IL-6 alteration in DRG and plasma of CCI and sham rats.

**Figure 9:**
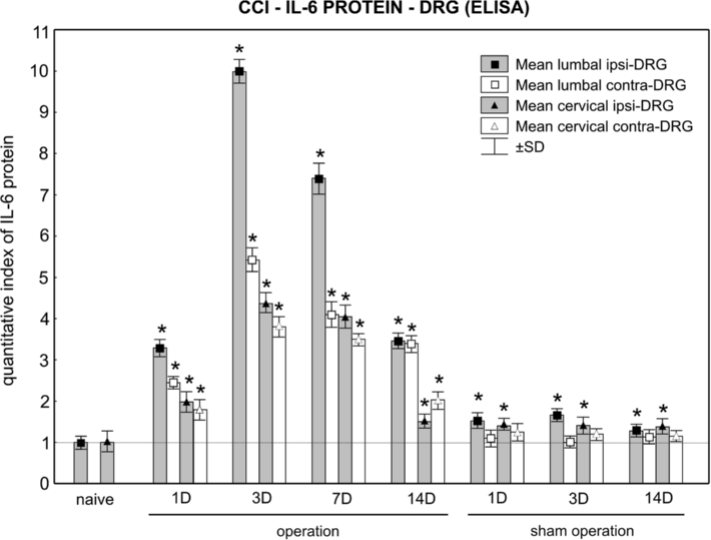
**ELISA of interleukin (IL)-6 protein in dorsal root ganglia (DRG).** Levels of IL-6 protein measured by ELISA in the ipsilateral (ipsi-DRG) and contralateral (contra-DRG) DRG of lumbar (L4-L5) and cervical (C7-C8) spinal segments removed from naive rats and rats after unilateral chronic constriction injury (CCI) of the sciatic nerve or sham operation. *Significant difference (*P*<0.05) when the protein levels of individual times of survival were compared with the baseline level of naive rats.

Compared with DRG of naive rats, increased levels of IL-6 protein were found bilaterally in both lumbar and cervical DRG from 1 to 14 days after unilateral CCI of the sciatic nerve. The levels of IL-6 protein peaked on day 3, when they were 10 and more than 5 times higher in ipsilateral and contralateral lumbar DRG, respectively. These levels declined afterwards, but remained higher than those of naive DRG (Figure 9).

Significant increases in IL-6 levels were found in ipsilateral lumbar and cervical DRG from sham rats surviving for 1, 3, and 14 days. The corresponding contralateral DRG did not display significant elevation of IL-6 protein compared with naive DRG (Figure 9).

**Figure 10:**
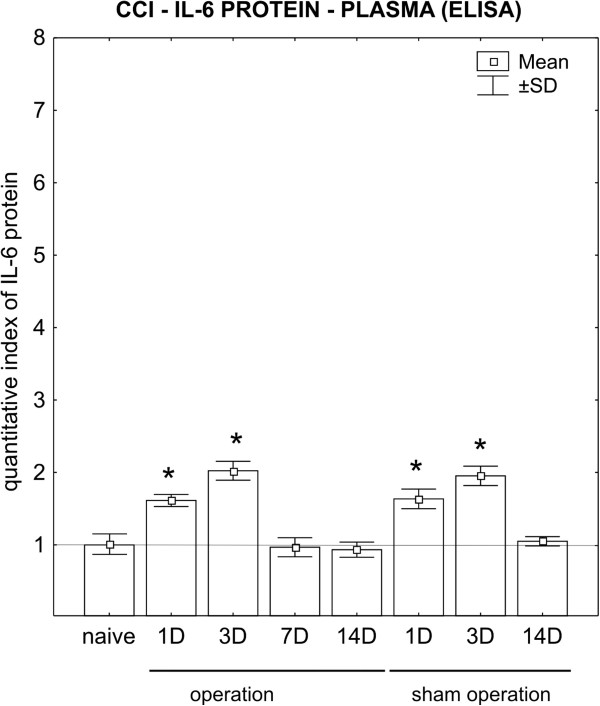
**ELISA of interleukin (IL)-6 protein in plasma.** Levels of IL-6 protein measured by ELISA in plasma of the same animals as for dorsal root ganglia (DRG). *Significant difference (*P*<0.05) when the protein levels for individual times of survival were compared with the baseline levels of naive rats.

The IL-6 protein levels increased approximately 1.5 and 2 times in the plasma of CCI and sham rats surviving for 1 and 3 days, respectively. Levels of plasma IL-6 protein then normalized during the time to the next survival time points (Figure 10).

### Western blot analysis

**Figure 11:**
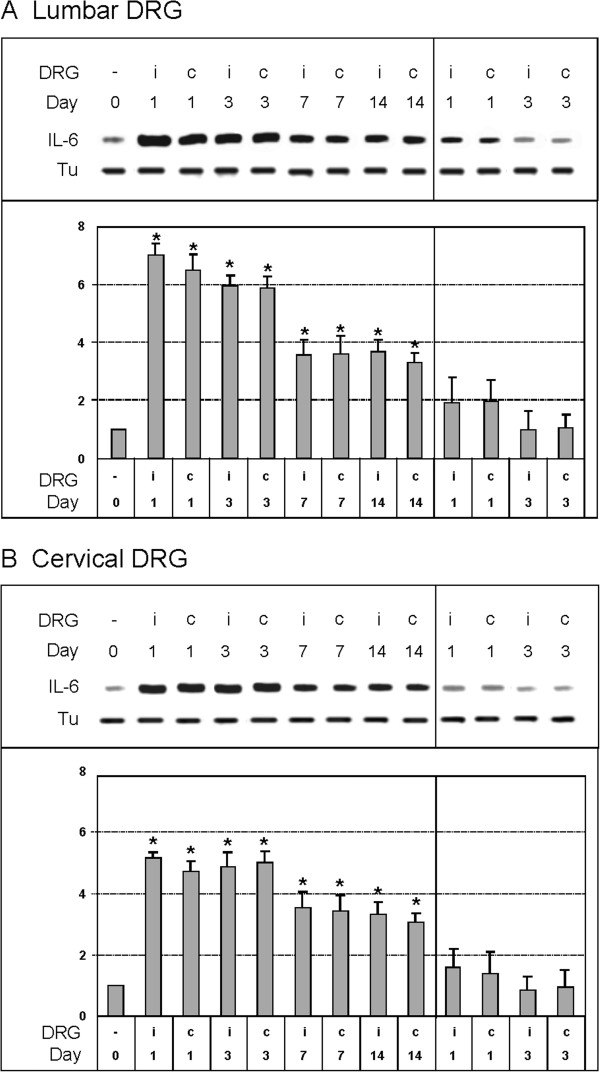
**Western blot analysis of interleukin (IL)-6 protein levels.** Western blot analysis of IL-6 protein levels in dorsal root ganglia (DRG) at (**A**) L4-L5 and (**B**) C7-C8 from naive rats (0), rats with unilateral chronic constriction injury (CCI) of the sciatic nerve after 1, 3, 7, and 14 days, and rats with sham surgery after 1 and 3 days. Upper panels illustrate representative western blotting bands of ipsilateral (I) and contralateral (C) DRG from two naive, two CCI, and two sham rats for each period of survival. Equal loading of proteins was confirmed by α-tubulin staining (Tu). Lower panels show mean density ± SD of individual IL-6 protein bands in triplicate analysis after normalization to tubulin, with the mean density of the IL-6 bands from naive DRG taken as 1.

Western blot analysis showed bilateral increases in IL-6 protein levels in both lumbar (Figure 11A) and cervical DRG (Figure 11B) of CCI rats compared with DRG of naive DRG. Densitometry of western blots for total L4-L5 DRG fractions showed a significant bilateral increase in IL-6 protein at 1, 3, 7, and 14 days after surgery. At 1 and 3 days after CCI, the levels of IL-6 protein were increased bilaterally by about 6 (L4-L5 DRG) and 5 (C7-C8 DRG) times in comparison with DRG of the naive rats. The levels of IL-6 protein remained similarly increased, by more than 3 times, in DRG of both lumbar and cervical segments at postoperative days 7 and 14.

Compared with naive rats, sham rats had higher levels of IL-6 protein in both lumbar and cervical DRG removed 1 day after the sham operation. The IL-6 protein levels in DRG from sham rats surviving for 3 days were similar to those of naive rats.

### *In situ* hybridization

**Figure 12:**
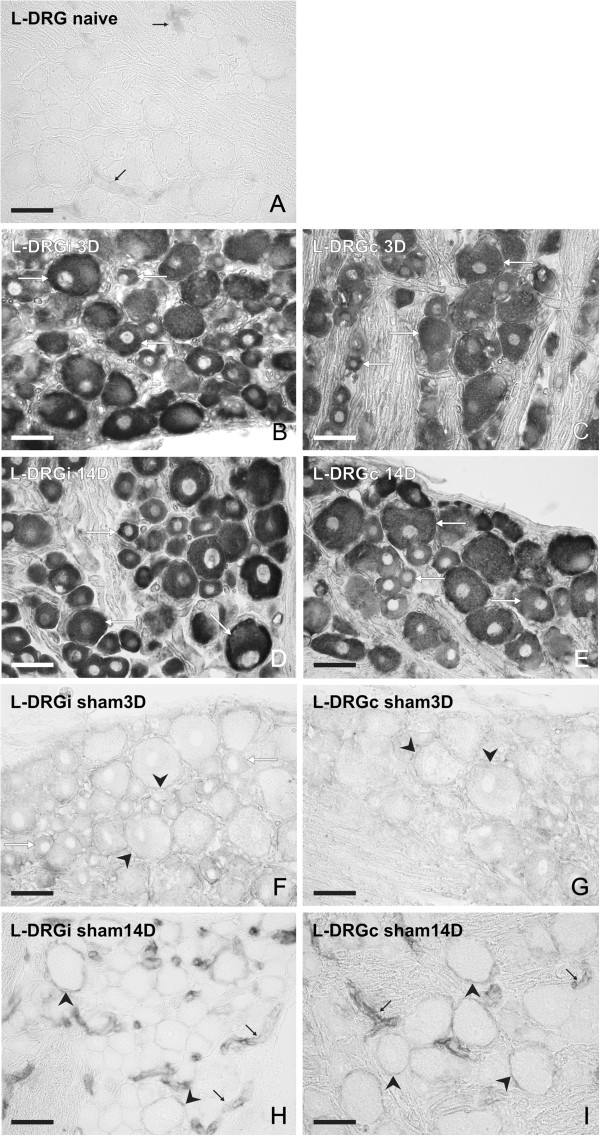
***In situ *hybridization for interleukin (IL)-6 mRNA in lumbar dorsal root ganglia (DRG).** Representative sections of *in situ* hybridization for IL-6 mRNA in (**A**) lumbar DRG (L-DRG) from naive rat, and in (**B–I)** ipsilateral (L-DRGi) and contralateral (L-DRGc) DRG from (**B–E**) rats with chronic constriction injury (CCI) at (**B,C**) 3 and (**D,E**) 14 days after surgery, and (**F–I)** sham-operated rats (**F,G**) 3 and (**H,I**) 14 days after surgery. Staining for IL-6 mRNA was present in blood vessels (black arrows), neuronal bodies of various sizes (white arrows), and satellite glial cells (SGC; arrowheads). Scale bars = 50 μm.

**Figure 13:**
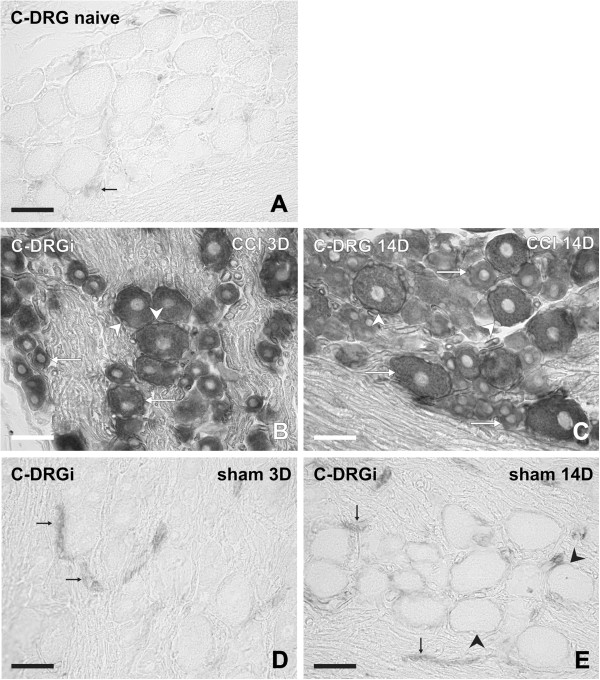
***In situ *hybridization for interleukin (IL)-6 mRNA in cervical dorsal root ganglia (DRG).** Representative *in situ* hybridization staining for IL-6 mRNA in sections through cervical DRG (C-DRG) from (**A**) naive rat, (**B,C**) rat with chronic constriction injury (CCI) at (**B**) 3 and (**C**) 14 days after surgery, and sham-operated rat at (**D**) 3 and (**E**) 14 days after surgery. Staining for IL-6 mRNA was present in blood vessels (black arrows), neuronal bodies of various sizes (white arrows), and satellite glial cells (SGC; arrowheads). Scale bars = 50 μm.

Neuronal bodies and their SGC displayed no or very weak signal for IL-6 mRNA in sections of lumbar and cervical DRG removed from naive rats. Faint staining for IL-6 mRNA was detected only in the blood vessels of naive DRG (Figure 12A, Figure 13A). Unilateral CCI of the sciatic nerve induced conspicuous bilateral elevation of staining for IL-6 mRNA in both lumbar and cervical DRG 1, 3, 7, and 14 days after operation. The patterns and intensities of staining for IL-6 mRNA were very similar in lumbar and cervical DRG sections for 1 and 3 days and for 7 and 14 days of survival, and hence representative illustrations from 3 and 14 days are shown (Figure 12B–E, Figure 13B,C). In comparison with naive DRG, an increased intensity for IL-6 mRNA staining was seen, particularly in neuronal bodies of all sizes and in the SGC surrounding the large neurons. No detectable signal for IL-6 mRNA was found in cells of the DRG capsule, but there was staining lining the Schwann cells of nerve fibers inside the DRG.

**Figure 14:**
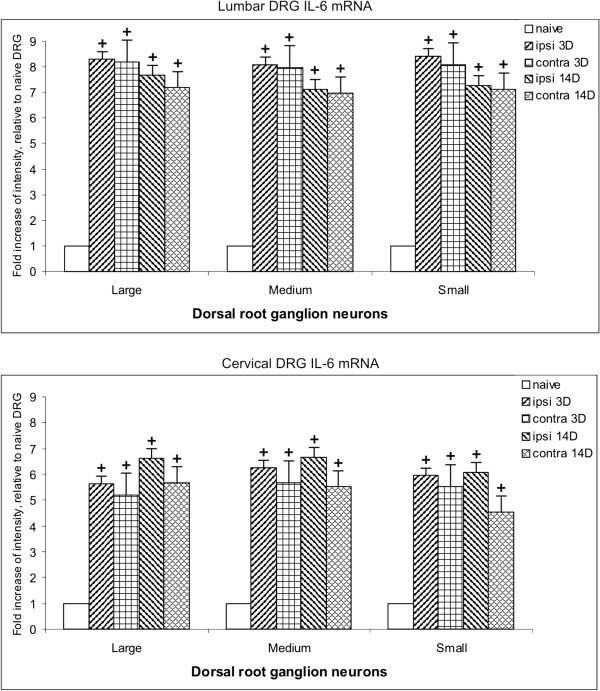
**Results of interleukin (IL)-6 mRNA staining density in dorsal root ganglia (DRG).** Results of staining density for IL-6 mRNA in lumbar (L-DRG) and cervical (C-DRG) dorsal root ganglia removed from naive rats (naive) and those subjected to chronic constriction injury (CCI) for short (3 days) and late (14 days) periods of survival. Measurement by image analysis system was carried out in large, medium, and small neuronal bodies of ipsilateral (ipsi) and contralateral (contra) DRG. +Significant difference (*P*<0.05) compared with naive rats.

When the DRG sections were treated for *in situ* hybridization under the same conditions, the density of IL-6 mRNA staining was higher in sections of ipsilateral than contralateral lumbar DRG for short (1 and 3 days) and long (7 and 14 days) periods of survival (Figure 14). The intense staining for IL-6 mRNA in neuronal bodies did not usually allow staining to be distinguished in the thin layer of the SGC envelope. This was possible only in some SGC surrounding the large neuronal bodies and mainly in DRG from sham rats (Figure 12F–I; Figure 13E).

Compared with lumbar DRG, the sections of cervical DRG from CCI rats had weaker staining for IL-6 mRNA in neuronal bodies, and therefore the staining of SGC was highlighted (Figures 13B,C). The density of IL-6 mRNA staining was slightly higher in medium and small neurons for short (1 and 3 days) than for long (7 and 14 days) periods of survival. No marked differences in IL-6 mRNA staining were seen between ipsilateral and contralateral cervical DRG (Figure 14).

In comparison with naive DRG, stronger staining for IL-6 mRNA was seen in SGC and small neuronal bodies of both ipsilateral and contralateral lumbar DRG removed after 3 days from sham rats. Increased staining intensity was seen in SGC and blood vessels but no neuronal staining was found in lumbar DRG of rats surviving 14 days after sham operation (Figure 12F–I). Although at 3 days after sham operation, the cervical DRG on both sides displayed increased staining for IL-6 mRNA in blood vessels only, a weak signal was found in SGC when DRG were removed 14 days after sham operation (Figure 13D–E).

## Discussion

IL-6 is a multifunctional cytokine whose increased level in the nervous system is rapidly and strongly induced by injury and by pathological and inflammatory stimuli [28]. A very low level of IL-6 was reported in the peripheral nervous system of intact mature animals, but its increase is induced distal to the site of sciatic nerve injury [29,30] and in the large and medium lumbar DRG neurons [15,31]. A weaker IL-6 induction was found in a CCI model of neuropathic pain, but it persisted for a longer time than in a nerve transection model. IL-6 induction in the CCI model was shown to correlate well with the duration of hypersensitivity [16]. It was reported that intrathecally administered human recombinant IL-6 elicited touch-evoked hyperalgesia in normal rats [32] and an injection of IL-6 into the rat hind paw induced dose-dependent mechanical hyperalgesia [33]. These findings suggest that IL-6 may be centrally involved in the cascade of events leading to the development of neuropathic pain. However, in addition to cytokines, upregulation of other immune mediators such as chemokines may also increase excitation of the DRG neurons in reaction to nerve injury [34,35].

### Cellular localization of interleukin-6 protein and mRNA in dorsal root ganglia

In agreement with previous papers [30,36], our results for immunohistochemical staining and *in situ* hybridization show that the primary sensory neurons are significant sources for enhanced IL-6 in DRG after CCI of the sciatic nerve. As indicated by ATF-3 staining, increased IL-6 expression was not limited to neurons with axonal injury but was also present in all neurons of DRG. In particular, significant enhancement of IL-6 immunostaining was clearly visible in large neuronal bodies, with greater enhancement in lumbar DRG ipsilateral to CCI than in contralateral DRG. The neuronal bodies of all sizes in cervical DRG and the medium and small neurons of lumbar DRG had increased IL-6 immunostaining but without significant differences between ipsilateral and contralateral sides. Double immunostaining showed that SGC, macrophages, and T cells may also contribute to the enhanced level of IL-6 protein in DRG.

Very little or no IL-6 protein or mRNA were detected in DRG neurons of sham rats, indicating that intraneuronal occurrence of IL-6 protein and its synthesis is induced by unilateral CCI of the sciatic nerve. Intraneuronal IL-6 mRNA and protein have been detected in DRG after various types of nerve manipulation [15,31,37]. In contrast to previous reports of induction of IL-6 mRNA only in the medium-sized and large lumbar DRG neurons [15,31], we found distinct increases in signal for IL-6 mRNA in DRG neurons of all sizes. This discrepancy is possibly due to the different *in situ* hybridization methods used, with radiographic probes being used by Murphy and coworkers (35S-labeled or 33P-labeled oligonucleotides) but a non-radioactive method (DIG-labeled probes) in our experiments. An *in situ* hybridization method with DIG-labeled probes can be used successfully to detect mRNAs in frozen sections with a sensitivity equal to or better than that of a radioactive method but with a much higher cellular resolution [38]. Therefore, an *in situ* hybridization method with DIG-labeled probes was used successfully in our experiments to detect IL-6 mRNA in neuronal bodies and their SGC in frozen DRG sections removed from rats.

It is well-known that SGC are activated by nerve injury, and they may also play a role in the development of pathological pain [39]. Compared with naive DRG, there was higher intensity of immunofluorescence for IL-6 and of staining for IL-6 mRNA in SGC of lumbar and cervical DRG of both sides after CCI or sham operation. However, it was difficult to distinguish unequivocally between IL-6 mRNA staining in neuronal bodies and their SGC of lumbar DRG, because a similar staining intensity was present in both cell types. Detection of IL-6 mRNA in SGC was easier in sections of cervical DRG when the staining density of neuronal bodies was lower than in lumbar DRG. Moreover, in contrast to neurons, SGC of both lumbar and cervical DRG removed from sham rats displayed increased staining for IL-6 mRNA, thus indicating that their activation and resultant synthesis of IL-6 may occurre only in response to tissue damage during surgical treatment. These results of activated SGC in DRG of sham rats correspond with findings of SGC proliferation and activation in response to scarification or incision of the skin [40,41].

IL-6 acts by binding to IL-6R and activating the gpl30 transducer chain. Although IL-6R expression is limited in cells of the nervous system [42], we found bilateral increases in IL-6R immunostaining in all neurons of both lumbar and cervical DRG from CCI rats. However, only SGC of lumbar DRG ipsilateral to CCI of the sciatic nerve displayed distinct immunostaining for IL-6R. This indicates some small differences of IL-6 action in DRG directly associated and not associated with injured nerve [43].

### Bilateral expression of interleukin-6 protein and mRNA in dorsal root ganglia after unilateral chronic constriction injury of the sciatic nerve

Bilateral expression of IL-6 protein and mRNA in DRG was not unexpected, because there is a growing body of evidence that unilateral nerve damage results in bilateral changes in neurochemical and electrophysiological parameters in DRG [44-46], including cytokines [18,37,47,48]. It has been generally accepted that contralateral responses to unilateral nerve injury are usually qualitatively similar but smaller in magnitude and have a briefer time course compared with ipsilateral changes [49]. However, our results showed that levels of IL-6 protein and mRNA in the contralateral DRG paralleled those of the ipsilateral DRG, not only in homonymous but also in heteronymous spinal cord segments with injured nerve. Bilateral upregulation of IL-6 in lumbar and cervical DRG after unilateral nerve injury is comparable with the expression of tumor necrosis factor (TNF)-α and IL-10 [50,51]. This indicates that cytokine upregulations in DRG that are associated or not associated with damaged nerve are induced by similar mechanisms.

The original CCI method using chromic gut [52] is a widely applied experimental model that induces characteristic signs and symptoms of neuropathic pain found in humans. However, because chromic gut itself induces local inflammatory reaction, this original CCI model of neuropathic pain is not suitable for distinguishing neuroinflammatory reactions induced by a thread material and/or Wallerian degeneration of injured axons [53,54]. Therefore, we prepared CCI of the sciatic nerve in our experimental rats using 3-0 sterilized suture (Ethicon) under aseptic conditions. Thus, the bilateral changes in IL-6 protein and mRNA in both lumbar and cervical DRG presented here were largely induced by partial traumatic nerve injury accompanied by neuroinflammatory response of Wallerian degeneration.

Possible mechanisms of contralateral signaling were reviewed by Koltzenburg and coworkers [49], but the underlying molecular mechanisms and neuroanatomical pathways linked with bilateral DRG responses to unilateral peripheral nerve injury remain largely unknown. Two main types of stimuli may be involved in inducing bilateral changes in IL-6 protein and mRNA in both lumbar and cervical DRG after unilateral CCI of the sciatic nerve. The first type of stimuli could be transferred by neuronal pathways, for example, through interneurons at the spinal cord and supraspinal levels [55-57]. Moreover, there is a long ascending propriospinal system linking lumbar and cervical spinal-cord segments. The so-called long ascending propriospinal neurons are defined as interneurons whose somata are located in the lumbar spinal-cord segments and whose axons terminate in cervical segments. These neurons are in an anatomically appropriate position to participate in coordinating movements of hind and fore limbs [58]. Changes in neuronal activity may partially contribute to the induction of IL-6 expression in neurons [59], although other mechanisms of IL-6 regulation, for example by systemic factors, can be assumed because DRG do not contain a complete blood–nerve barrier [60].

These other possible mechanisms inducing IL-6 mRNA and protein in DRG are probably related to production of signaling molecules during Wallerian degeneration. Experimental findings have suggested that IL-6 is induced in DRG by an injury factor arising from the nerve stump rather than by interruption of retrograde axonal transport of signal molecules from target tissues or distal nerve segments [15,16]. The results from the current study implicate Wallerian degeneration as one possible source of the factors inducing bilateral increases in IL-6 mRNA and protein in both lumbar and cervical DRG after unilateral CCI performed under aseptic conditions. The type of signal molecules produced by Wallerian degeneration can pass through an interrupted blood–nerve barrier [61,62], thus allowing diffusion of circulating signal molecules into the microenvironment of the DRG not associated with the injured nerve. Several candidate molecules have been suggested for signaling from damaged nerve, including ATP, glutamate, complement or prostaglandin E2 [63-66]. Some of these signal molecules are probably produced by damaged tissue during surgical treatment, as was indicated by induction of IL-6 mRNA and protein in SGC of DRG removed from sham rats.

ATP, which is suggested to be one of the first mediators of tissue damage, acts through ionotropic P2X receptors and metabotropic P2Y receptors [67]. It has been shown that the P2X3 receptor of DRG neurons and the P2X7 receptor expressed in SGC are two major purinergic receptors participating in neuron–SGC communication. The bodies of excited DRG neurons release ATP and activate P2X7 receptor, which is expressed only in SGCs [68-70]. Although it is noteworthy that P2X7 receptor is associated with inflammatory reactions of SGC in order to induce their sensitivity to ATP, and that this may significantly contribute to neuropathic pain [71], it has been shown that activation of P2X3 contributing to mechanical hyperalgesia does not depend on pro-inflammatory cytokines, including IL-6 [72]. However, the P2X7 receptor is upregulated in human DRG and injured nerves obtained from patients with chronic neuropathic pain. It has been reported that P2X7 receptor knockout animals did not display mechanical or thermal hyperalgesia although normal nociceptive processing was preserved [73]. However, involvement of the P2X7 receptor in the regulation of IL-6 is controversial. It was reported that ATP-induced IL-6 production is not mediated by P2X7 receptors [74], whereas others found markedly increased levels of IL-6 in inflamed hind paw of P2X7^-/-^ mice [73], indicating that this receptor is involved in regulating IL-6 levels. This might be present in the case of SGC, which, in contrast to DRG neurons, express both P2X7 [73] and IL-6.

### Serum level of interleukin-6 protein after chronic constriction injury of the sciatic nerve

There are some controversial results illustrating changes in plasma IL-6 after rat nerve injury and the involvement of these changes in neuropathic pain induction. It was reported that an increase in plasma IL-6 had no effect on pain [14], whereas other evidence suggested that such an increase had a hyperalgesic effect [33]. In our experiments, the plasma IL-6 protein was increased approximately 1.5 and 2 times in rats surviving 1 and 3 days from CCI and sham operation, respectively. By contrast, the increase in IL-6 protein in DRG was significantly higher (3 to 10 times), thus suggesting that plasma IL-6 did not contribute substantially to the increase of the cytokine in DRG. A decrease in IL-6 to the level seen in naive rats 7 and 14 days after CCI, when mechanical hyperalgesia and thermal hyperalgesia were measured, indicates that changes in plasma IL-6 induced by nerve injury do not correlate exactly with induction and maintenance of neuropathic pain.

In the context of neuropathic pain, it is important to mention that pro-inflammatory cytokines, of which IL-6 is the main one, also induce behavioral discomfort or ‘sickness’, not only in response to infection but also after nerve trauma [75,76]. Although the level of IL-6 protein in blood plasma has specifically been shown to be correlated with infection-induced sickness [75], its level in neuropathic pain status is controversial [14,33,77]. The animals in our experiments were housed under specific pathogen-free conditions, and behavioral tests were measured under conditions that would minimize stress on the animals.

The level of plasma IL-6 in both CCI and sham rats was increased only at 1 and 3 days of survival, and normalized thereafter. A higher level of plasma IL-6 during the short period of survival corresponds with published findings [78], and reflects a post-operation reaction that is probably related to bilateral thermal hyperalgesia in both hind and fore paws of sham-operated rats.

Our behavioral tests are in accordance with published results [79] showing that sham surgery without nerve manipulation is sufficient to induce temporary hyperalgesia, as also measured by some other authors [80,81]. Bilateral thermal hyperalgesia was identical in both hind and fore paws of sham rats in our experiments, thus indicating that effects of surgery treatment are adequate to alter mechanisms of sensory processing in both fore and hind paws. However, the same pattern of thermal hyperalgesia was not seen in sham versus CCI rats. To explain these findings, we believe that the central control structures may be differently activated after sham and CCI treatment of animals and thus would have resulted in their different behavioral reactions to heat stimuli. The sham operation probably did not activate central control mechanisms, as these are triggered by nerve injury [82-84].

### A possible functional involvement of interleukin-6 protein in dorsal root ganglia associated or not associated with injured nerve

IL-6 may be critically involved in the cascade of events after nerve injury leading to the development and maintenance of behaviors suggestive of neuropathic pain. Fundamental evidence as to a role of IL-6 in nociception and hyperalgesia has been found by direct injection of IL-6 into experimental animals [14,32]. In addition, IL-6^-/-^ mice showed reduced heat sensitivity in a hot-plate behavioral test [85]. Our results confirmed the increase in IL-6 mRNA and protein in DRG neurons ipsilateral to CCI for at least 14 days when hypersensitivity was apparent in the ipsilateral hind paws [16]. This indicates that continuous upregulation of IL-6 in DRG associated with injured nerve is linked to induction of hyperalgesia. Different animal neuropathic pain models have been established to cover the diverse etiology and consequently the diverse clinical manifestation of neuropathic pain, with high validation and reproducibility. However, they have shortcomings and limitations that should be taken into consideration. Principally, the measured alterations in cutaneous sensory thresholds might be responses to nerve injury rather than integrated pain-related behavior reactions. Moreover, because for ethical reasons experimental animals usually survive only days or weeks, the clinical aspects of neuropathic pain are measured in years [86]. Thus, animal models are relevant predominantly for testing of induction of neuropathic pain, and have limited value for simulating chronic hypersensitivity changes in patients.

Our results from immunohistochemistry, western blotting, and *in situ* hybridization unequivocally showed an unexpected IL-6 response in contralateral lumbar and cervical DRG on both sides, which did not coincide with behavioral signs of hypersensitivity in corresponding paw skin. A similar pattern of expression was found for TNF-α and IL-10 [50,51] suggesting that these changes in DRG occurring after unilateral CCI may reflect a general neuroinflammatory reaction of the nervous system to injury. Involvement of cytokines upregulated in remote DRG with neuropathic pain induction is still unknown, and other experiments are required to elucidate this.

Elevation of IL-6 in DRG that are not associated with injured nerves indicates a possible functional involvement of IL-6 other than that of neuropathic pain induction. Several lines of evidence have shown that IL-6 is implicated as a key component in the injury response of the nervous system. Consistent with our findings for IL-6, there is evidence that nerve growth factor mRNA is also increased bilaterally in lumbar and cervical DRG after unilateral crushing of the sciatic nerve [87]. Moreover, IL-6 plays a role in promoting neuronal survival [12] and axonal growth by DRG neurons [37]. Thus, IL-6 upregulation in the primary sensory neurons of DRG that are not associated with damaged nerve might be linked with conditioning of the intact neurons to regenerate their axons [88,89].

## Conclusion

In a rat model of neuropathic pain based on aseptic unilateral CCI of the sciatic nerve, we found that the contralateral L4-L5 DRG and the cervical DRG on both sides were not spared from IL-6 response to unilateral sciatic nerve injury even though these DRG were not directly linked with the damaged nerve. The increase in IL-6 protein and mRNA in the ipsilateral lumbar DRG only was related to (or at least coincided with) behavioral presentations of hyperalgesia in the corresponding limb.

Although these findings of increased IL-6 protein and mRNA in DRG associated with damaged nerve may support a role for IL-6 in developing neuropathic pain, the finding of IL-6 in DRG not associated with injured nerve argues against a direct coupling between IL-6 elevation in DRG and hypersensitivity. The results of our IL-6 study suggest that neuroinflammatory reaction of DRG to nerve injury is propagated alongside neuroaxis from lumbar to remote cervical segments. This phenomenon probably illustrates a general neuroinflammatory reaction of the nervous system to local nerve injury.

## Competing interests

The authors declare that they have no competing interests.

## Authors’ contributions

PD conceived, designed and coordinated the study and wrote the manuscript. VB conceived, designed, and coordinated the western blot and *in situ* hybridization analyses. IK and IS conceived, designed and carried out the experiments, and participated in acquiring and analyzing the presented data. All authors gave final approval to the version to be published.
